# Metabolomic Insights of the Effects of Bacterial Algicide IRI-160AA on Dinoflagellate *Karlodinium veneficum*

**DOI:** 10.3390/metabo12040317

**Published:** 2022-04-01

**Authors:** Yanfei Wang, Kathryn J. Coyne

**Affiliations:** School of Marine Science and Policy, College of Earth, Ocean & Environment, University of Delaware, 1044 College Drive, Lewes, DE 19958, USA; yfwang@udel.edu

**Keywords:** *Shewanella*, *Karlodinium veneficum*, dinoflagellate, algicide, programmed cell death, DNA damage, reactive oxygen species, metabolomics

## Abstract

*Shewanella* sp. IRI-160 is an algicidal bacterium that secretes an algicide, IRI-160AA. This algicide specifically targets dinoflagellates, while having no adverse effects on other algal species tested. Dinoflagellates exposed to IRI-160AA exhibited increased production of reactive oxygen species (ROS), DNA damage, and cell cycle arrest, implying a programmed pathway leading to cell death (PCD). Here, a metabolomic analysis was conducted on dinoflagellate *Karlodinium veneficum* and a control cryptophyte species *Rhodomonas* exposed to IRI-160AA to investigate the cellular mechanisms behind the physiological effects and the specificity of this algicide. Results of this research supported previous observations about physiological responses to the algicide. A suite of metabolites was identified that increased in the cell pellets of *K. veneficum* but not in *Rhodomonas*, including oxidative stress biomarkers, antioxidants, and compounds involved in DNA damage and PCD. Overall, the results of this study illustrated the metabolomic mechanisms underlying the algicidal effects of IRI-160AA on dinoflagellates. This research also provided insights and future directions for studies on the cellular response of dinoflagellates exposed to antagonistic bacteria in the environment.

## 1. Introduction

Phytoplankton are responsible for almost half of the photosynthesis on earth and play essential roles in regulating global biogeochemical cycles [1,2,3]. The microbes surrounding and/or associated with phytoplankton are also key players in this process; heterotrophic bacteria recycle up to 90% of organic matter derived from phytoplankton [4]. Interactions between phytoplankton and bacteria in the aquatic ecosystems are diverse and complex [5,6]. These interactions include mutualism, commensalism, antagonism, parasitism, and competition, governed by the metabolites and other chemicals exchanged by these microbes [3]. Relationships between phytoplankton and bacteria are also dynamic in that interactions can be altered by the physical state of these microbes [3].

Dinoflagellates are among the most dominant groups of phytoplankton in the marine ecosystem, and are the second largest group of primary producers in the ocean, behind diatoms [7]. They also form important symbiotic relationships with other organisms, such as reef-building corals [8]. Nevertheless, dinoflagellates can also cause massive and persistent harmful algal blooms (HABs) that pose a threat to other organisms in the environment [9]. In fact, dinoflagellates are among the most toxigenic HAB species [9]; they are able to produce a wide range of compounds that can be bioaccumulated through the food web to cause disease and death of marine organisms and negatively impact human health [10,11]. The HAB dinoflagellate *Karlodinium veneficum* is a mixotrophic species that has caused harmful algal blooms worldwide for decades [12]. This organism can produce a suite of toxic compounds, termed karlotoxins, that are hemolytic, ichthyotoxic, and cytotoxic [12]. Karlotoxins can decrease the grazing rates of predatory phytoplankton and zooplankton, increasing the survival of *K. veneficum* in the environment [13,14]. These toxins have also been associated with massive fish kills worldwide [15].

It has been demonstrated that bacteria surrounding and/or associated with dinoflagellates play a critical role in the biology and ecology of these algal species. For instance, research demonstrated that some dinoflagellates are vitamin B1 and B12 auxotrophs [16] and rely on their bacterial partners to provide these micronutrients [17]. In addition to the beneficial effects, several species of bacteria have shown algicidal activity against dinoflagellates that can either kill or inhibit the growth of these algal species [4,18,19,20,21,22]. Algicidal bacteria may exhibit specific activity against dinoflagellates [21], while others also affect the growth of other species [18,19]. Two modes of action have been involved in the activity of algicidal bacteria: these bacteria can either attach to the targeted algal species and attack them directly, or they may secrete algicidal compounds to control the growth of targeted algal species indirectly [4,22]. For instance, two strains of bacteria designated S03 and 41-DBG2 both exhibited algicidal effects on the dinoflagellates *Karenia brevis* and *Karenia mikimotoi* [20]. While S03 required direct contact to lyse the cells, 41-DBG2 could kill these dinoflagellates by secreting water-soluble compounds [20]. Little is known about algicidal compounds produced by bacteria, but those that have been identified are diverse, spanning a wide range of structures, polarity, and size [4].

*Shewanella* sp. IRI-160 is an algicidal bacterium isolated from the Delaware Inland Bays, DE, USA [21]. This bacterium can secrete water-soluble algicidal compounds, collectively referred to as IRI-160AA, that specifically inhibit the growth of dinoflagellates, including *K. veneficum* [23]. No negative effects have been observed upon exposure of other algal species, zooplankton, invertebrates, or juvenile finfish to IRI-160AA [21,23,24]. Research on *K. veneficum* physiology after exposure to IRI-160AA demonstrated induction of reactive oxygen species (ROS), DNA damage, and cell cycle arrest, along with caspase-like protease activity, suggesting that the algicide induces a programmed pathway leading to cell death (PCD) [25,26]. Studies on the photobiology of *K. veneficum* and other dinoflagellates exposed to IRI-160AA also indicated disruption of photosynthetic electron transport [27]. Preliminary analysis demonstrated that the algicidal fraction is polar [23], and identified ammonium among the active algicidal compounds in IRI-160AA [28]. There were significant differences, however, between exposure to IRI-160AA produced by *Shewanella* vs. ammonium alone in terms of dinoflagellate photobiology [29] and gene level regulation [30]. Recent transcriptomics studies of *K. veneficum* exposed to IRI-160AA revealed a global response to the algicide, including processes affecting gene expression, protein activity, and cellular morphology [30]. Genes involved in ROS response, DNA damage, and PCD were also differentially expressed in *K. veneficum* exposed to IRI-160AA compared to controls where the algicide was not added [30]. Amines, including the polyamine putrescine, have also been identified as algicidal components of IRI-160AA [28]. A negative synergistic effect was reported by ammonium and putrescine on dinoflagellates including *K. veneficum*, and molecular data indicated ammonium may disrupt polyamine homeostasis, while putrescine may sensitize these species to ammonium toxicity [30].

Recent improvements in metabolomics analysis now provide researchers with an opportunity to explore algae–bacteria interactions at a systemic level. For example, recent studies used metabolomics analysis in co-cultivation systems to investigate the effects of bacteria on algae [31,32], and on bacterial algicides produced under different environmental conditions [33]. A metabolomics study of diatom *Thalassiosira pseudonana* co-cultured with bacterium *Dinoroseobacter shibae* exhibited an upregulation of various critical pathways involving intracellular amino acids compared to the algal monocultures [32], suggesting an adaptive response of the diatom to the presence of this bacterium [32]. Other research also investigated changes in the metabolomics profile of cyanobacteria exposed to multiple chemical algicides, including copper sulfate, hydrogen peroxide, and sodium carbonate peroxide [34]. So far, no research has been conducted to investigate changes in the metabolome by algal species in response to bacterial algicides.

In this research, metabolomics analysis was conducted on dinoflagellate *K. veneficum* exposed to IRI-160AA to explore the effects of this algicide at the metabolomics level. Metabolomics analysis of a non-target cryptophyte species, *Rhodomonas* sp., was also conducted as a control to elucidate mechanisms underlying the specificity of this algicide for dinoflagellates. Finally, an exometabolomics approach distinguished those metabolites that were present in the cell pellet from those that may have been introduced to the culture by the algicide itself. In addition to a number of diverse processes related to ammonium metabolism and detoxification in both species, results of this study identified a suite of metabolites that was upregulated in *K. veneficum* but not *Rhodomonas*. Metabolites that were specific to *K. veneficum* included biomarkers for oxidative stress, antioxidants, as well as vitamins and metabolites known to induce DNA damage and participate in PCD in other organisms, supporting previous research that algicidal compounds in IRI-160AA may act synergistically with ammonium to induce cell death in dinoflagellates.

## 2. Results

**Figure 1:**
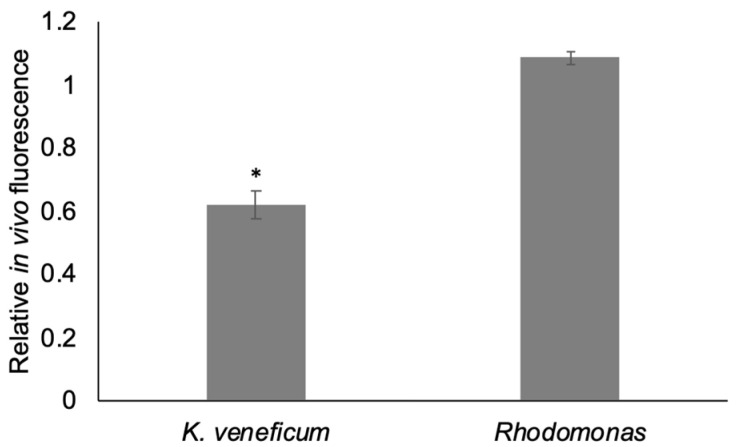
In vivo fluorescence of *Karlodinium veneficum* and *Rhodomonas* sp. in the algicide treatments (with IRI-160AA) relative to controls after 19 h of exposure. Error bars indicate standard deviations of 3 replicates. Asterisks indicate a significant difference of relative in vivo fluorescence of the indicated treatments compared to the controls (*p* < 0.05).

The in vivo fluorescence (as a proxy for cell density; [23]) of *K. veneficum* treated with the algicide was significantly lower (61.78%) than the control after 19 h exposure (*p* < 0.05; Figure 1 and Figure S1). No significant difference in in vivo fluorescence was observed between the treatment and the control for *Rhodomonas* sp. (*p* > 0.05).

**Figure 2:**
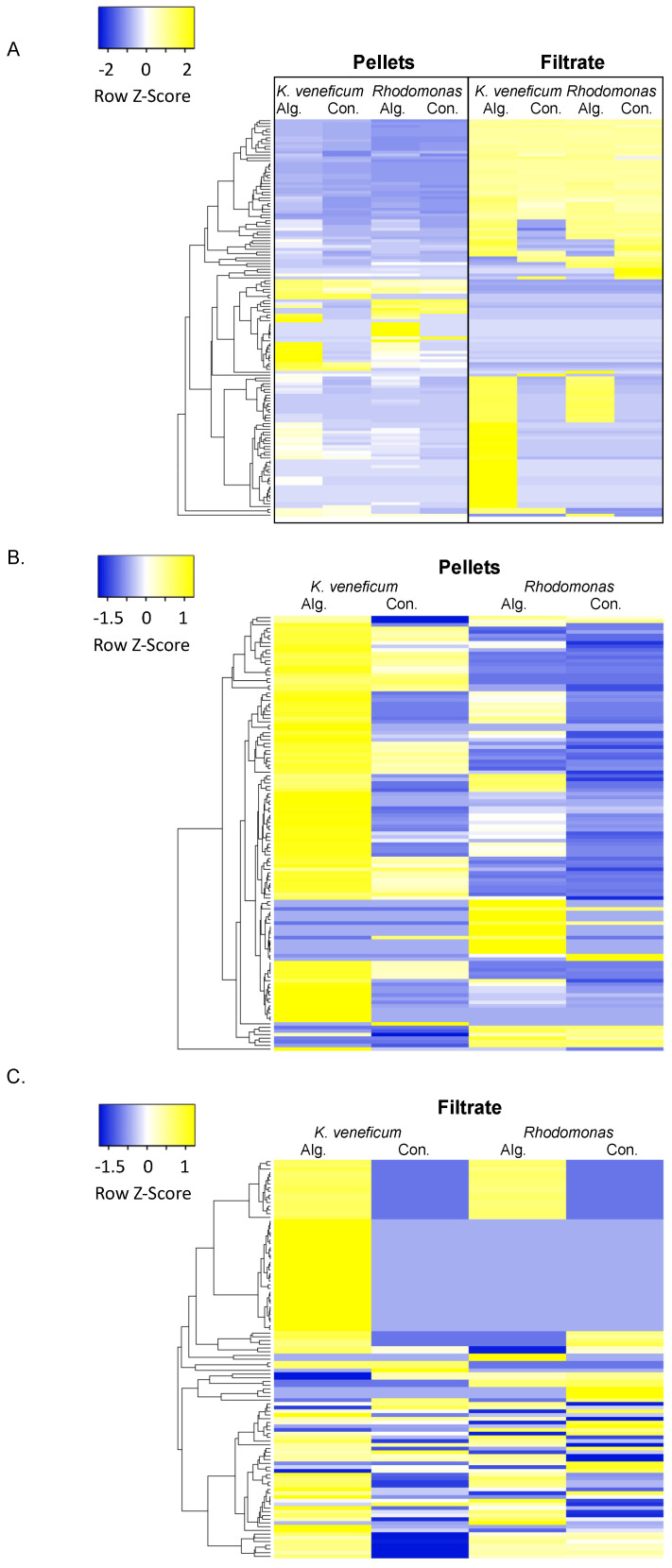
Heatmaps of the annotated compounds in the cell pellets and filtrate of *Karlodinium veneficum* and *Rhodomonas* treated with the algicide (Alg.) compared to the control (Con.; with no algicide addition). All annotated compounds were included in the heatmap. (**A**) Heatmap of all annotated compounds; (**B**) heatmap of compounds identified in the cell pellets; (**C**) heatmap of the compounds identified in the cell filtrate. Relative concentrations of each compound (RC) were log10 (RC+1) transformed first, and the row z-scores represent the normalized log10 (RC+1) across each row. The heatmap was created using Heatmapper [35].

The metabolomics analysis detected 1140 polar and semi-polar compounds in the samples, and a total of 139 compounds that were above the detection limits across all samples were annotated (Table S1, Figure 2A). Among these annotated compounds, 122 were identified in the cell pellets, and 107 were in the cell filtrate (Figure 2B,C).

Only the annotated compounds with a relative concentration altered by the algicide addition by at least 1.5-fold were selected for further analysis. In this dataset, 93 compounds were detected in the *K. veneficum* cell pellets and 71 compounds were detected in the *K. veneficum* cell filtrate samples that exhibited higher relative concentrations in the treatment compared to the control. For *Rhodomonas*, the relative concentrations of 51 compounds were higher in the treatment compared to the control in the cell pellets, and 33 metabolites had higher relative concentrations in the treatment compared to the control for the cell filtrate.

Additionally, three metabolites had lower relative concentrations in the treatment compared to the control in the cell pellets and six in the filtrate of *K. veneficum*. Four compounds had lower relative concentrations in the treatment compared to the control in the *Rhodomonas* cell pellet and twenty-four in the filtrate samples.

### 2.1. Metabolomics Profile of Cell Pellets

**Figure 3:**
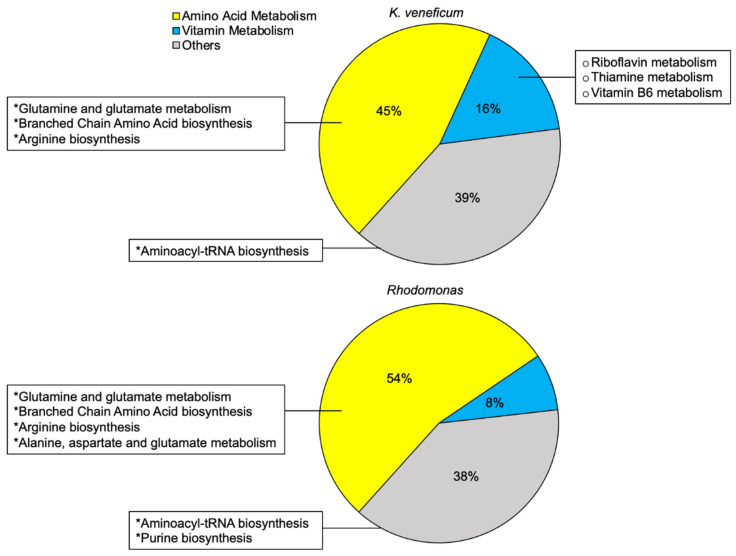
Metabolites that increased in the cell pellets in the algicide treatment compared to the control (>1.5-fold) were mapped to 31 KEGG pathways in *Karlodinium veneficum* and 26 pathways in *Rhodomonas* [36]. A. The percentage of amino acid and vitamin metabolism in the mapped pathways, as well as other processes among these mapped pathways in each species (calculated as the number of pathways in categories/number of all pathways × 100%). “*” indicates the significance of the metabolite sets’ enrichment of the indicated pathway (FDR < 0.05). “°” indicates the vitamin metabolism pathways that were only mapped by the increased metabolites in the cell pellets stimulated by the algicide compared to the control for *K. veneficum* but not *Rhodomonas*. See Figure S2 for the detailed enrichment analysis.

A total of 74 compounds that exhibited higher relative concentrations in the algicide treatment compared to the control were included for the metabolite enrichment analysis for *K. veneficum* cell pellets (Figure 3 and Figure S2). These compounds were mapped to 31 KEGG pathways, with almost half of these pathways involved in amino acid metabolism (45%). Vitamin metabolism contributed an additional 16% to all mapped pathways. Four pathways were enriched by these compounds (FDR < 0.05), including the aminoacyl-tRNA biosynthesis, glutamine and glutamate metabolism, as well as biosynthesis of branched chain amino acids (BCAA; valine, leucine, and isoleucine) and arginine. Though not significantly enriched, the metabolite sets’ enrichment revealed the potential involvement of B vitamin metabolism (riboflavin, thiamine, and pyridoxal) in *K. veneficum* cell pellets treated with the algicide (FDR > 0.05).

Forty-three compounds that had higher relative concentrations in the algicide treatment compared to the control were included in the metabolite sets’ enrichment analysis for *Rhodomonas* sp. cell pellets (Figure 3 and Figure S2). These compounds were mapped to 26 KEGG pathways. Similar to the enrichment analysis for *K. veneficum* cell pellets, the majority of these mapped pathways belong to amino acid metabolism (54%). Vitamin metabolism contributed 8% of all mapped pathways, but notably, the pathways involved in the metabolism of riboflavin, thiamine, and pyridoxal that had been identified for *K. veneficum* cell pellets were not observed here. These metabolites were enriched in six pathways (FDR < 0.05), including three pathways shared with *K. veneficum* (aminoacyl-tRNA biosynthesis, metabolism of glutamate and glutamine, and biosynthesis of BCAA), as well as metabolism of purine nucleotides and biosynthesis of alanine and aspartate.

The compounds that had lower relative concentrations in the algicide treatment compared to the control were not included in the metabolite sets’ enrichment analysis due to the low numbers of compounds.

### 2.2. Metabolites Related to Vitamins

**Figure 4:**
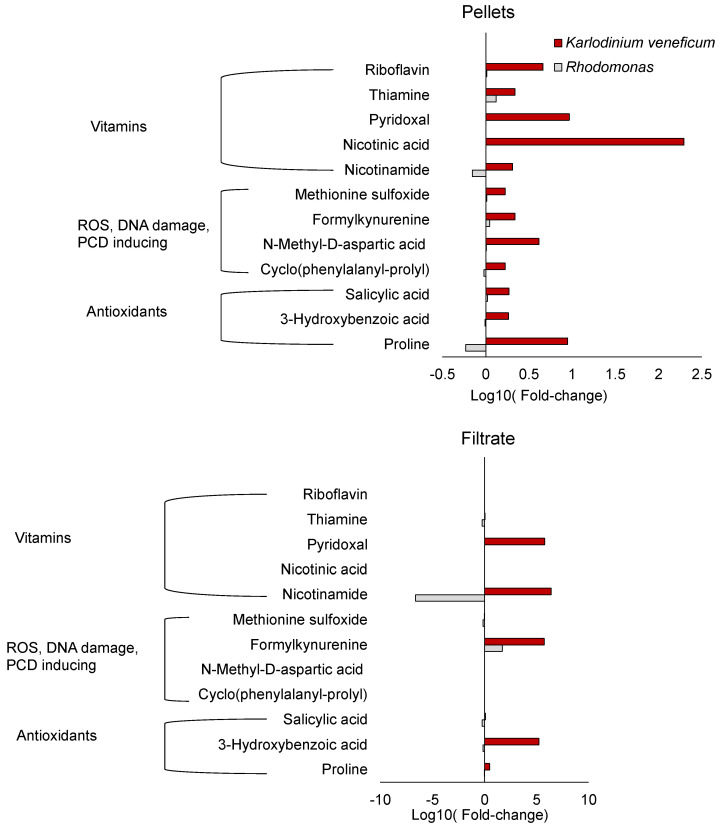
Relative changes in B vitamins, metabolites involved in ROS production, DNA damage, and/or PCD, and antioxidants that increased in the cell pellets of *Karlodinium veneficum* but not *Rhodomonas*. Figures represent the log10 transformed fold-change of relative concentrations of these compounds in the cell pellets (**upper**) and the filtrate (**lower**) of the algicide treatment over the control (with no algicide addition). The analysis was based on the averaged peak areas (normalized to cell densities for the cell pellets) of duplicate samples; error bars were not included.

Several B vitamins, including riboflavin, thiamine, pyridoxal, nicotinic acid, and nicotinamide, increased in the cell pellets of *K. veneficum* treated with the algicide compared to the control (Figure 4). None of these vitamins were enriched in cell pellets of *Rhodomonas*. Pyridoxal and nicotinamide increased in the cell filtrate of *K. veneficum* treatment as well, while the other B vitamins were not enriched in the cell filtrate of this species. In *Rhodomonas* cell filtrate, thiamine decreased in algicide treatments while nicotinamide increased in concentration compared to the control.

### 2.3. Metabolites Related to ROS Production, DNA Damage, and Programmed Cell Death

Five compounds that could be potentially involved in ROS production, DNA damage, and/or PCD were identified: methionine sulfoxide (MetO), formylkynurenine (*N*′-formylkynurenine; NFK), *N*-methyl-d-aspartic acid (NMDA), cyclo(phenylalanine-proline) (cFP), and phenylalanine (Figure 4; phenylalanine not shown). MetO, NMDA, and cFP only increased in the cell pellets of the algicide treatment of *K. veneficum* but not their filtrate compared to the control. The relative concentrations of these compounds did not change in the cell pellets or filtrate of the algicide treatment of *Rhodomonas*. NFK increased in the cell pellets of *K. veneficum* but not *Rhodomonas* exposed to the algicide compared to the respective controls. It also increased in the cell filtrate of both *K. veneficum* and *Rhodomonas* algicide treatments compared to the respective controls. Phenylalanine increased in cell pellets and filtrate of the treatments for both *K. veneficum* and *Rhodomonas* compared to the respective controls.

Antioxidants, including salicylic acid (SA), 3-hydroxybenzoic acid (3-HBA), and proline increased only in the cell pellets of the algicide treatment of *K. veneficum* compared to the control (Figure 4), while the levels of these compounds did not change (SA and 3-HBA) or decreased (proline) in the cell pellets of *Rhodomonas* treatment compared to the control. In the cell filtrate of *K. veneficum* treatment, the level of SA did not change while 3-HBA and proline increased compared to the control. In the cell filtrate of *Rhodomonas* treatment, the level of SA decreased, while concentrations of 3-HBA and proline did not change.

### 2.4. Ammonium Concentrations

Results indicated that the ammonium concentration in the stock algicide was 3.20 mM. At the initial time point, algicide was added to each of the treatment cultures at 4.97% (*v*/*v*; EC50) of the total volume, leading to a calculated addition of 159 μM ammonium in the treatment compared to the control (no algicide addition).

**Figure 5:**
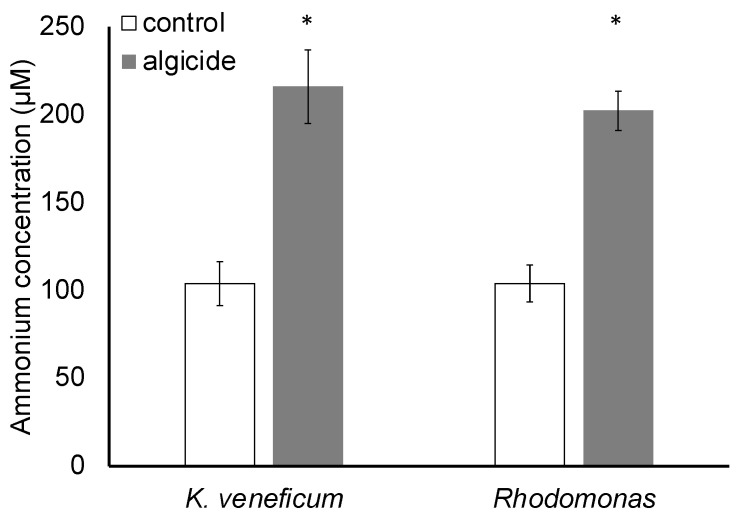
Ammonium concentrations in *Karlodinium veneficum* and *Rhodomonas* in controls (with no algicide addition) and the algicide treatment measured after 19 h incubation. Error bars indicate standard deviations of 3 replicates. Asterisks indicate a significant difference of ammonium concentrations in the indicated treatment compared to the control (*p* < 0.05).

After 19 h, significant differences were observed in ammonium concentrations between the treatments and controls of both species (*p* < 0.05; Figure 5). Ammonium concentrations in the treatment and control of *K. veneficum* were 216 and 104 μM, respectively, and 203 and 104 μM in the *Rhodomonas* treatment and control, respectively. No significant differences in ammonium concentrations between the two species were observed in the control or the treatment (*p* > 0.05).

## 3. Discussion

Previous research demonstrated that the algicide IRI-160AA produced by *Shewanella* sp. IRI-160 specifically targets dinoflagellates [21,23]. Exposure to the algicide impaired photosynthesis, increased ROS production, and induced DNA damage and cell cycle arrest, along with other markers indicating a programmed pathway leading to cell death (PCD) [25,26,27]. Molecular evidence supporting these physiological effects was also identified in transcriptomics analysis [30]. Research on IRI-160AA indicated that ammonium was among the active algicidal compounds, and that other compounds in the algicide contributed to the observed algicidal effects [28]. In this study, a metabolomics approach was used to identify changes in metabolites for the dinoflagellate *K. veneficum* after exposure to IRI-160AA compared to control cultures. Metabolomics analysis was also conducted on a non-target control species, the cryptophyte *Rhodomonas* sp., to elucidate mechanisms behind the selectivity of IRI-160AA for dinoflagellate species.

**Figure 6:**
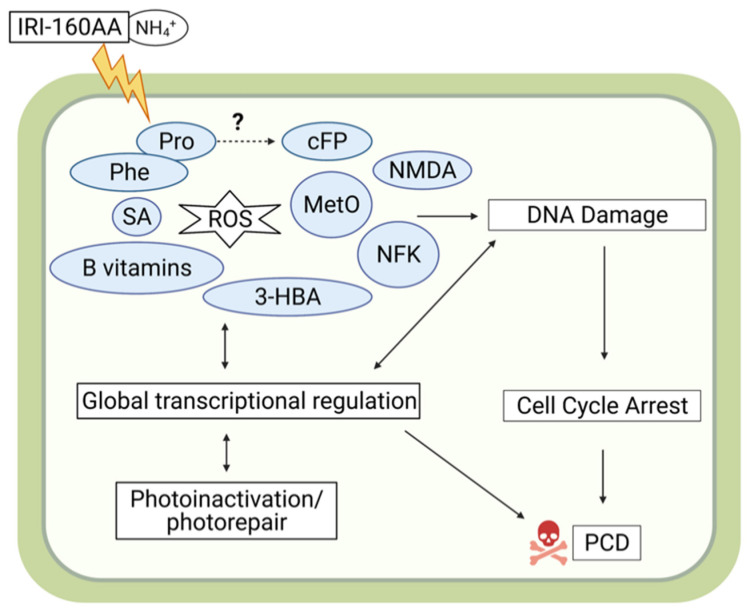
Schematic representation of a model depicting the cellular impact of IRI-160AA on *Karlodinium veneficum* revealed by this study and previously reported molecular and physiological data [25,26,27,30]. In this model, IRI-160AA stimulated ROS production, inducing the formation of oxidative stress markers, methionine sulfoxide (MetO), and formylkynurenine (*N*′-formylkynurenine; NFK) in the cell pellets of the algicide treatment of *K. veneficum*. Cellular antioxidants also increased in response to ROS, including salicylic acid (SA), 3-hydroxybenzoic acid (3-HBA), proline, and B vitamins (riboflavin, thiamine, pyridoxal, nicotinic acid, and nicotinamide). Other compounds indicative of reactive oxygen species (ROS), DNA damage, and programmed cell death (PCD) that increased in the cell pellets included *N*-methyl-d-aspartic acid (NMDA), phenylalanine, and cyclo(phenylalanine-proline) (cFP). cFP can enter the cells by simple diffusion, or by the biosynthesis from phenylalanine and proline, where the pathway has not been fully characterized (indicated by “?” in the figure). The figure was created with BioRender.com.

Results of bioassays (Figure 1) were consistent with previous studies showing the specificity of the algicide IRI-160AA to dinoflagellates, including *K. veneficum* [21,23]. The differences in response to the algicide were supported by metabolomics analysis, in which a suite of compounds was found to increase only in the cell pellets of *K. veneficum* but not *Rhodomonas* when exposed to the algicide (Figure 4 and Figure 6). These compounds included antioxidants, vitamins, and other metabolites that can potentially contribute to oxidative stress, DNA damage, and PCD, highlighting the specific effects of IRI-160AA on dinoflagellates in relationship with previously observed physiological responses [25,26,30]. 

Previous research observed higher intra- and extracellular ROS production in dinoflagellates exposed to the algicide IRI-160AA compared to the control [25], suggesting the potential for increased levels of antioxidants and oxidation products in cells exposed to the algicide. Accumulation of metabolites that serve as biomarkers for oxidative stress has been observed in plants [37,38] and algae [39,40,41]. One such marker, MetO, is the oxidation product from methionine, the most susceptible amino acid to ROS oxidation [42,43]. MetO has been used as a reliable biomarker for oxidative stress as well as an indicator for ineffective repair mechanisms [42].

The identification of MetO in this study is consistent with previous reports demonstrating ROS production and induction of PCD in dinoflagellates exposed to IRI-160AA [25] (Figure 4 and Figure 6). MetO inactivates proteins, and detoxification of this compound through the reduction of MetO by MetO reductase is crucial to alleviate the adverse effects of ROS [43]. For example, MetO reductase mutants of plants are associated with an increase in ROS concentrations and induction of PCD [43]. Administration of MetO to animals through subcutaneous injection also induced DNA damage and apoptosis, accompanied by increased ROS and antioxidant enzyme activities [44]. The selective accumulation of MetO in the cell pellets of *K. veneficum* treated with IRI-160AA compared to controls in this study implies a conserved role of MetO among different kingdoms of organisms. The accumulation of MetO in *K. veneficum* but not in *Rhodomonas* also suggests that *K. veneficum* was not able to efficiently detoxify this compound.

Similar to MetO, formylkynurenine (*N*′-formylkynurenine; NFK) is an oxidative stress biomarker and also an amino acid oxidation product [45,46,47]. NFK is a post-translational modification of tryptophan that can be formed during the oxidation of its side-chain exposed to multiple ROS, including singlet oxygen, ozone, and hydroxyl radicals [45,46]. NFK has been identified as a biomarker for oxidative stress in plants exposed to high light, and may play a role in photosystem II (PSII damage and repair signaling [45,46]. Research has also suggested that NFK may serve as a protein antioxidant and protect mitochondria from oxidative stress [47]. The selective increase of NFK in the cell pellets of *K. veneficum* is consistent with the observed ROS production stimulated by the algicide [25] (Figure 4 and Figure 6). To be noted, NFK is also a photosensitizer; research has indicated its production may stimulate its further accumulation in the cells [47]. Here, the increase of NFK in the cell filtrate of both *Rhodomonas* and *K. veneficum* exposed to the algicide compared to the control implies NFK may also be (partially) provided by the algicide itself. However, the effect of extracellular NFK application is not clear and requires future research.

An increase in antioxidant levels also serves as a marker for oxidative stress [48,49]. In this study, there was an increase in antioxidant levels in the cell pellets of *K. veneficum* treated with IRI-160AA but not in *Rhodomonas* (Figure 4 and Figure 6). These antioxidants included salicylic acid (SA; 2-hydroxybenzoic acid), a ubiquitous phytohormone that plays a central role in the resistance and tolerance of plants in response to various abiotic and biotic stresses [50]. As an antioxidant, SA can either directly scavenge ROS or indirectly regulate the redox balance by enhancing antioxidant defense in plants [51,52], including increasing the activity of antioxidant enzymes [53]. Both intracellular accumulation and exogenous application of SA have been demonstrated to enhance antioxidant defense and tolerance to stresses in plants [51,53]. In plant cells, the concentration of SA may tip the balance from survival to death. For example, accumulation of SA at high concentrations at the microbial infection site in *Arabidopsis thaliana* triggered a local immune response, favoring PCD, while at low concentrations, SA induced a systemic immune response, enhancing cell survival at locations that were distant from the infection site [54]. As evidence has been provided for PCD in dinoflagellates exposed to the algicide IRI-160AA [25], the increased SA in the cell pellets of *K. veneficum* treatment compared to the control implied that this metabolite could serve a function similar to that observed in plants. A dose-dependent effect of SA on the growth of chlorophyte *Chlorella vulgaris* [55] and the cyanobacterium *Microcystis flos-aquae* [56] has also been demonstrated. The differential response of *K. veneficum* and *Rhodomonas* to the algicide may reflect their varied tolerance to SA, or species-specific SA requirements to cope with stresses induced by other compounds in IRI-160AA. Other studies have reported that extracellular application of SA increases the intracellular level of this compound [51,53]. Although there was a small increase of SA in the filtrate of *K. veneficum*, the decrease of SA in the cell filtrate of *Rhodomonas* suggests the potential for uptake of this compound if it was a component of the algicide.

Other antioxidants were also identified in *K. veneficum* cell pellets that were present in higher concentrations in treatments compared to the control (Figure 4 and Figure 6). The SA isomer 3-hydroxybenzoic acid (3-HBA), for example, exhibited antioxidant activities that could alleviate lipid peroxidation [57] and protect iron from autoxidation [58]. This metabolite was present in *K. veneficum* cell pellets as well as the culture filtrate of this species, but was not elevated for *Rhodomonas*. It is unclear if the increase of 3-HBA in the algicide treatment of *K. veneficum* was synthesized by this species in response to the oxidative stress caused by IRI-160AA [25], or was taken up from the algicide.

Proline was also enriched in *K. veneficum* cell pellets after exposure to the algicide (Figure 4 and Figure 6). This amino acid also has antioxidant properties [59,60]. Accumulation of proline is a common response of plants under a variety of stresses, where it plays a major role as a metal chelator, ROS scavenger, and signaling molecule [59,60]. Despite its beneficial role in stress tolerance, studies have also indicated that ROS production could be stimulated by both endogenous proline accumulation and exogenous proline application, leading to either cell survival or death, including through PCD, in a concentration-dependent manner [61]. The selective accumulation of proline in the cell pellets of *K. veneficum* exposed to the algicide was consistent with its role as an antioxidant, while higher concentrations may have played a role in initiating PCD in this species.

Other metabolites involved in DNA damage and PCD identified in this research included *N*-Methyl-d-aspartic acid (NMDA) and cyclo(phenylalanine-proline) (cFP); they were among the metabolites that only increased in the cell pellets of *K. veneficum* treatment compared to the control but not *Rhodomonas* (Figure 4 and Figure 6). NMDA is a known inducer of apoptosis in mammalian cells [62]; the apoptosis was accompanied by DNA nick and fragmentation [63]. Here, the increase of NMDA in the cell pellets of *K. veneficum* treated with the algicide compared to the control implied it may have a comparable role in dinoflagellates. Similar to the other metabolites that had a selective increase in the cell pellets of *K. veneficum* over *Rhodomonas* revealed in this research, its source (e.g., exogenous vs. endogenous) could not be identified with the current data.

Cyclo dipeptides (aka 2,5-diketopiperazines, CDPs) are ubiquitous compounds with prominent biological functions [64]. Among the CDPs, cyclo(phenylalanine-proline) (cFP; Figure 4 and Figure 6) is well-known for its quorum-sensing property in various bacterial species [65]. Though its function in algal species is not clear, cFP is able to pass biological membranes via simple diffusion [66] and induce apoptosis in mammalian cells [66], accompanied by increased caspase-3 activity [67], ROS production [68], and DNA damage [68]. cFP-induced apoptosis was mediated through the ATR-CHK2 pathway involved in the DNA damage response (DDR) [68,69]. The increase of cFP in *K. veneficum* cell pellets treated with the algicide compared to the control implied cFP could have a similar function in dinoflagellates. This was supported by previous transcriptomic data that suggested the possible involvement of an ATR-CHK2 DDR pathway in this species in response to IRI-160AA exposure [30].

Though cFP could have diffused into cells from IRI-160AA added to the culture medium [66], the selective increase of cFP contents in the cell pellets of *K. veneficum* over *Rhodomonas* implied the contribution of other sources, possibly the biosynthesis from phenylalanine and proline [65]. This was supported by the evidence that phenylalanine increased in the cell pellets of the algicide treatments of both *K. veneficum* and *Rhodomonas* compared to their respective controls (also indicated by the pathway enrichment analysis; Figure S2), while the intracellular proline only increased in the *K. veneficum* algicide treatment (Figure 4 and Figure 6).

B vitamins, including riboflavin (vitamin B2, [70]), thiamine (vitamin B1, [71]), pyridoxal (vitamin B6, [72]), nicotinic acid (vitamin B3, [73]), and nicotinamide (vitamin B3, [74]) are antioxidants that have essential protective roles in organisms. The increase of these B vitamins in the cell pellets of *K. veneficum* in the algicide treatment compared to the control (Figure 4 and Figure S2) may serve as additional biomarkers for oxidative stress, and their accumulation in cells of *K. veneficum* may help this species cope with the oxidative stress induced by the algicide [25]. The culture filtrate of algicide-treated *K. veneficum* was also enriched with pyridoxal and nicotinamide, suggesting the possibility that they were components of the bacterial filtrate IRI-160AA.

Comparison between the metabolomic profiles of the cell pellets of *K. veneficum* and the control cryptophyte *Rhodomonas* (Figure 2) also revealed a number of similar processes stimulated by the algicide (Figure 3 and Figure S2) that were likely related to the presence of ammonium in the algicide. The shared enriched processes included glutamine and glutamate metabolism, as well as biosynthesis of branched chain amino acids (BCAAs) and arginine (FDR < 0.05; Figure 3).

**Figure 7:**
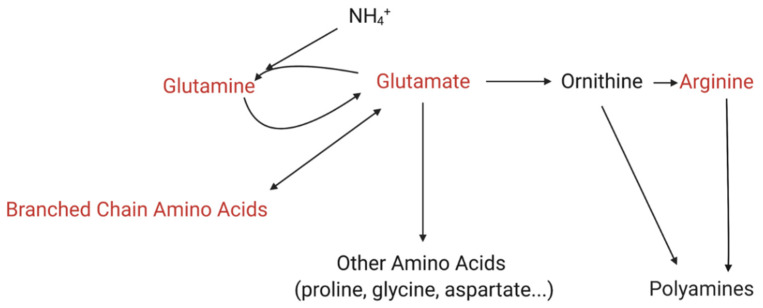
Simplified schematic demonstrating the relationship between ammonium assimilation and metabolism of glutamine, glutamate, branched chain amino acids, and arginine. These metabolites are indicated by a red font. Glutamate is the precursor of other amino acids in the cells, and a number of pathways related to their metabolism were identified in the enrichment analysis (see Figure 3 and Figure S2). The figure was created with BioRender.com.

Enrichment of glutamine and glutamate metabolism in both *K. veneficum* and *Rhodomonas* correlated with increased ammonium concentrations (Figure 5), introduced to the culture with the algicide. Glutamine synthetase/glutamate synthase (GS/GOGAT) pathway is the primary ammonium assimilation route in higher plants and algae, and plays an essential role in ammonium detoxification [75,76,77,78] (Figure 7). Glutamate produced here is the precursor for other nitrogen-rich metabolites, including proline, γ-aminobutyric acid (GABA), and arginine, among others [79]. Arginine can then be utilized to produce polyamines [79,80]. Nitrogen flow to polyamines has been identified as a major nitrogen sink for excess ammonium and is critical to alleviate ammonium toxicity [79,80]. In addition to the assimilation from ammonium, glutamate can also be produced from other sources in the cells, including from BCAA catabolism [81,82,83,84]. In fact, BCAAs are essential nitrogen donors for glutamate biosynthesis in animals, and play a critical role in ammonia detoxification through the production of glutamine from glutamate and ammonia [81,82]. The role of BCAAs in algae is not clear, while the pathways involved in BCAA’s role in ammonia detoxification are shared among animals, plants, and algae, implying a conserved function of these amino acids [81,82,83].

Comparison of ammonium concentrations for algicide-treated *K. veneficum* and *Rhodomonas* suggests similar uptake by these species (Figure 5), while metabolic analysis shows a similar response through ammonium detoxification pathways involving BCAAs, glutamine/glutamate, and arginine discussed above. Previous studies, however, have demonstrated that dinoflagellates have greater sensitivity to ammonium toxicity [85]. Polyamines present in the algicide [28] may also intensify the effects of ammonium on dinoflagellates [30]. A recent study demonstrated the synergistic negative effects of ammonium and putrescine on *K. veneficum*, and transcriptomic analysis revealed the role of polyamines by enhancing ammonium toxicity in *K. veneficum* [30].

Chemical exchanges between bacteria and algae in the microenvironment immediately surrounding an algal cell, termed the phycosphere, play an important role in algal growth and physiology (reviewed by [3]). Dinoflagellates and other algal taxa, for example, rely on bacterial communities to provide critical nutrients [86], and there is substantial evidence that bacteria–algal interactions have a significant effect on the growth of algae [87]. The choice to use non-axenic cultures in experiments to examine the effects of IRI-160AA on dinoflagellates is similar to other studies [23,25,26], and provides a more realistic and environmentally relevant understanding of dinoflagellate response to the algicide. Metabolomic analyses on algal species in non-axenic culture are also fairly common; for example, to evaluate metabolomic response due to allelopathic interactions between dinoflagellates and diatoms [88], to profile the metabolomic changes of diatoms during different growth stages [89], and other studies (see for example [90,91]). Similar to the rationale presented here, Vidoudez and Pohnert [89] pointed out that using non-axenic diatom cultures may better reflect metabolomic responses of diatoms in nature, and excluding bacterial communities in the cultures may cause additional stress that would influence the results. In the study presented here, the algal cell pellets were captured on filters, limiting the metabolomics analysis of the cell pellets to the algae and those bacteria that were attached to the cells, and eliminating free-living bacteria from the analysis. The limited effect of co-cultured bacteria on the algal cell pellets was evident by the fact that very few metabolites were shared by the cell pellets and filtrate from the same culture for either *K. veneficum* or *Rhodomonas*, even in the controls (Table S1). In summary, it was likely that bacterial cohorts in non-axenic cultures of *K. veneficum* or *Rhodomonas* made a (limited) contribution to the changes in the metabolomic profiles presented here, but the results obtained from analysis of non-axenic cultures are more representative of these species in the natural environment.

It should also be noted, this analysis only included polar and semi-polar metabolites. The metabolomic profiles of non-polar metabolites (e.g., lipids) of algae exposed to the algicide remain unknown. Furthermore, enriched metabolites within the algicide treatment compared to the control may also be from the addition of the algicide itself, although few of the enriched metabolites discussed here were found in both the cell pellet and the culture filtrate (Figure 4), and only one of these was also enriched in the culture filtrate from *Rhodomonas*. Instead, the stimulation of these compounds in the cell pellets by IRI-160AA likely reflects the cellular response of *K. veneficum* to the algicide. Future research should be directed to identify the sources of these metabolites through stable-isotope-labeling studies, for example.

In conclusion, this research identified the metabolomic response of dinoflagellate *K. veneficum* to the bacterial algicide IRI-160AA. A suite of metabolites was revealed to selectively increase in the cell pellets of *K. veneficum* but not *Rhodomonas* exposed to IRI-160AA. These metabolites included the oxidative stress biomarkers methionine sulfoxide and *N*′-formylkynurenine, a number of antioxidants (salicylic acid, 3-hydroxybenzoic acid, proline, and B vitamins), and compounds involved in DNA damage and PCD (*N*-methyl-d-aspartic acid and cyclo[phenylalanine-proline]). Pathways related to ammonium detoxification, including glutamine/glutamate metabolism, as well as biosynthesis of branched amino acids and arginine, were also upregulated in *K. veneficum*. This was consistent with the contribution of ammonium to the algicidal effect of IRI-160AA. At the same time, the observation that these processes were also upregulated in *Rhodomonas* implied that the specificity of IRI-160AA for dinoflagellates is not only related to the addition of ammonium, as noted in Ternon et al. [28]. This research provided insights into the algicidal effect of IRI-160AA on *K. veneficum* at the metabolomic level and expanded our knowledge of the metabolic response of dinoflagellates to bioactive substances produced by bacteria. Future research should be conducted to continue to identify the active algicidal compounds in the algicide IRI-160AA, and also explore the metabolomic effects of these compounds, individually or in combination, on dinoflagellates.

## 4. Materials and Methods

### 4.1. Algal Stock Culture

Non-axenic stock cultures of *Karlodinium veneficum* (CCMP 2936 [National Center for Marine Algae and Microbiota, Bigelow Laboratory for Ocean Sciences, East Boothbay, USA]; dinoflagellate) and *Rhodomonas* sp. (CCMP 757; cryptophyte) were maintained in natural seawater with f/2 nutrients (-Si) [92] and a salinity of 20, at 25 °C, and with a light intensity of approximately 130 µmol photons m^−2^ s^−1^. The cultures were kept under a 12 h:12 h light:dark cycle and semi-continuously in the exponential growth phase.

### 4.2. Algicide Preparation

IRI-160AA was prepared as described previously [23] with slight modification. Briefly, a single colony of *Shewanella* IRI-160 was transferred to LM medium [93]. The culture was incubated at 25 °C with shaking overnight at 100 rpm. The *Shewanella* IRI-160 culture was centrifuged at 6000 rpm for 5 min. The supernatant was discarded, and the cell pellet was resuspended in f/2 medium (-Si) [92]. The suspension was centrifuged again as above. The supernatant was discarded, and cell pellets were resuspended in f/2 medium (-Si) [92] and incubated at room temperature for 7 days. To prepare the cell-free algicide, the culture was filtered through a 0.2 µm nylon syringe filter (Corning, Corning, NY, USA).

### 4.3. Ammonium Concentration

Ammonium concentration in the algicide was measured using an API^®^ Ammonia Test Kit (Mars Fishcare Inc., Chalfont, PA, USA). The method was modified from the manufacturer’s instructions and was described previously [94]. Briefly, each of 250 μL hypochlorite solution and salicylate/catalyst solution was added to the 2.5 mL diluted sample and incubated at room temperature for 10 min. The absorbance of the sample at 690 nm was measured using a NanoDrop 2000 Spectrophotometer (ThermoFisher Scientific, Waltham, MA, USA). A standard curve was made with a series of diluted ammonium standards (Sigma-Aldrich St. Louis, MO, USA). The ammonium concentration in the sample was then calculated by linear regression analysis.

### 4.4. Metabolomic Response of K. veneficum and Rhodomonas sp. to Algicide IRI-160AA

#### 4.4.1. Culture Treatments

Algicide IRI-160AA was prepared as described above, and EC50 (half-maximal effective concentration) was determined on *K. veneficum* cultures (*N* = 3; 4.97% [*v*/*v*]). IRI-160AA at the EC50 level was added to 50 mL cultures of *K. veneficum* and *Rhodomonas* sp. in 250 mL polycarbonate Erlenmeyer flasks (*N* = 3). The control cultures received the same amount of f/2 medium without algicide (*N* = 3). After 19 h, in vivo fluorescence was measured for each culture. Microscopy cell counts were also performed (data not shown). The relative in vivo fluorescence of the treatment was calculated as: [in vivo fluorescence of the treatment/average in vivo fluorescence of the control] [23].

#### 4.4.2. Sample Preparation for Metabolomics Analysis

Cell pellets were collected by filtering each culture through GF/F filters (Whatman, Maidstone, UK). Metabolites were extracted from the filters using cold 60% methanol (*v*/*v* with MilliQ water) immediately after filtration. The mixture was vortexed for 30 s, and the filters were discarded. The culture filtrate was further filtered through 0.2 μm nylon syringe filters (Corning). The extracted cell pellets and culture filtrate were lyophilized, and duplicates of each sample were sent to MS-Omics (Vedbæk, Denmark) on dry ice for analysis.

#### 4.4.3. Sample Analysis for Polar and Semi-Polar Metabolites

Metabolomics analysis was carried out by MS-Omics using a Vanquish LC coupled to Thermo Q Exactive HF MS (Thermo Fisher Scientific, Waltham, MA, USA). An electrospray ionization interface was used as an ionization source. The analysis was performed in negative and positive ionization mode. Ultra Performance Liquid Chromatography (UPLC) was performed for semi-polar [95] and polar [96] compounds using slightly modified versions of the protocols.

Peak areas were extracted using Compound Discoverer 2.0 (Thermo Fisher Scientific). Annotation was performed at four levels; Level 1: identification by retention times (compared against in-house authentic standards), accurate mass (with an accepted deviation of 3 ppm), and tandem mass spectrometry (MS/MS) spectra; Level 2a: identification by retention times (compared against in-house authentic standards) and accurate mass (with an accepted deviation of 3 ppm); Level 2b: identification by accurate mass (with an accepted deviation of 3 ppm) and MS/MS spectra; Level 3: identification by accurate mass alone (with an accepted deviation of 3 ppm).

#### 4.4.4. Data Analysis

Averaged peak areas of compounds detected in the cell pellets were normalized to the cell density of each species at the sampling time point. The normalized peak areas were considered as relative concentrations (RC) of each compound. A heatmap was constructed using Heatmapper [35] from the log10 (RC+1) transformed values of compounds that were annotated (including all annotation levels).

The fold-change of each compound comparing the treatment and control was calculated as: [Average peak area (treatments) + 1]/[Average peak area (controls) + 1]. Only compounds that were altered by the algicide by at least 1.5-fold (>1.5 to call “increase” or <0.67 to call “decrease”) were included in the metabolite enrichment analysis using MetaboAnalyst [36] with the KEGG pathway database and the default settings. The compounds that could not be matched in the MetaboAnalyst pathway library and metabolite set database were filtered out [36]. The pathways with an FDR < 0.05 were considered enriched.

Additionally, antioxidants and metabolites related to ROS production, DNA damage response, and programmed cell death that increased in the cell pellets of *K. veneficum* but not *Rhodomonas* were identified by searching the literature. The same thresholds, >1.5-fold and <0.67-fold changes, were used to consider a compound to be increased or decreased, respectively, in the treatment compared to the control.

### 4.5. Statistical Analysis

One-way ANOVA was used to evaluate the significant differences in the relative in vivo fluorescence or ammonium concentrations between the controls and treatments of *K. veneficum* and *Rhodomonas* (*p* < 0.05). The same test was also conducted to assess the significant differences in ammonium concentrations of the controls or the algicide treatments between these species (*p* < 0.05).

## 5. Conclusions

This investigation provided insights into the metabolic responses of two phytoplankton species after exposure to algicide IRI-160AA, produced by naturally-occurring marine bacterium *Shewanella* sp. IRI-160. Results of this research expanded our knowledge of the effects of microbially-produced algicides on algal taxa with varying sensitivities, and revealed underlying metabolic changes in response to these bioactive substances. The suite of metabolites that was selectively increased in dinoflagellate *Karlodinium veneficum* included several biomarkers for oxidative stress, and a number of antioxidants, as well as compounds involved in DNA damage and PCD. Future research may be directed to determination of the synergistic effects of compounds identified in IRI-160AA on the dinoflagellate metabolome.

## Data Availability

All metabolomics data for this research can be found in the Supplementary Materials (Table S1).

## Supplementary Materials

The following supporting information can be downloaded at: https://www.mdpi.com/article/10.3390/metabo12040317/s1, Table S1: Log10 (RC+1) transformed relative concentrations (RC) of compounds detected in the cell pellets and filtrate of *Karlodinium veneficum* and *Rhodomonas*; Figure S1: In vivo fluorescence (FLR) of the harmful dinoflagellate *Karlodinium veneficum* and the control cryptophyte *Rhodomonas* treated with f/2 medium without the algicide (control) or the algicide for 19 h. Error bars represent standard deviations of 3 replicates. “*” indicates significant differences between the control and the treatment (*p* < 0.05); Figure S2: Metabolite sets’ enrichment analysis on metabolites increased (>1.5-fold comparing the treatment and the control) in cell pellets of *Karlodinium veneficum* (A) and *Rhodomonas* sp. (B).
