# Supplementary material for: Metabolomic Insights of the Effects of Bacterial Algicide IRI-160AA on Dinoflagellate Karlodinium veneficum
Source: Metabolites. 2022 Apr 1;12(4):317. doi: 10.3390/metabo12040317 (PMC9030264; doi:10.3390/metabo12040317)
Supplement: Supplementary file 1 [file metabolites-12-00317-s001.zip › metabolites-1664955 Supplementary Figures.pdf]

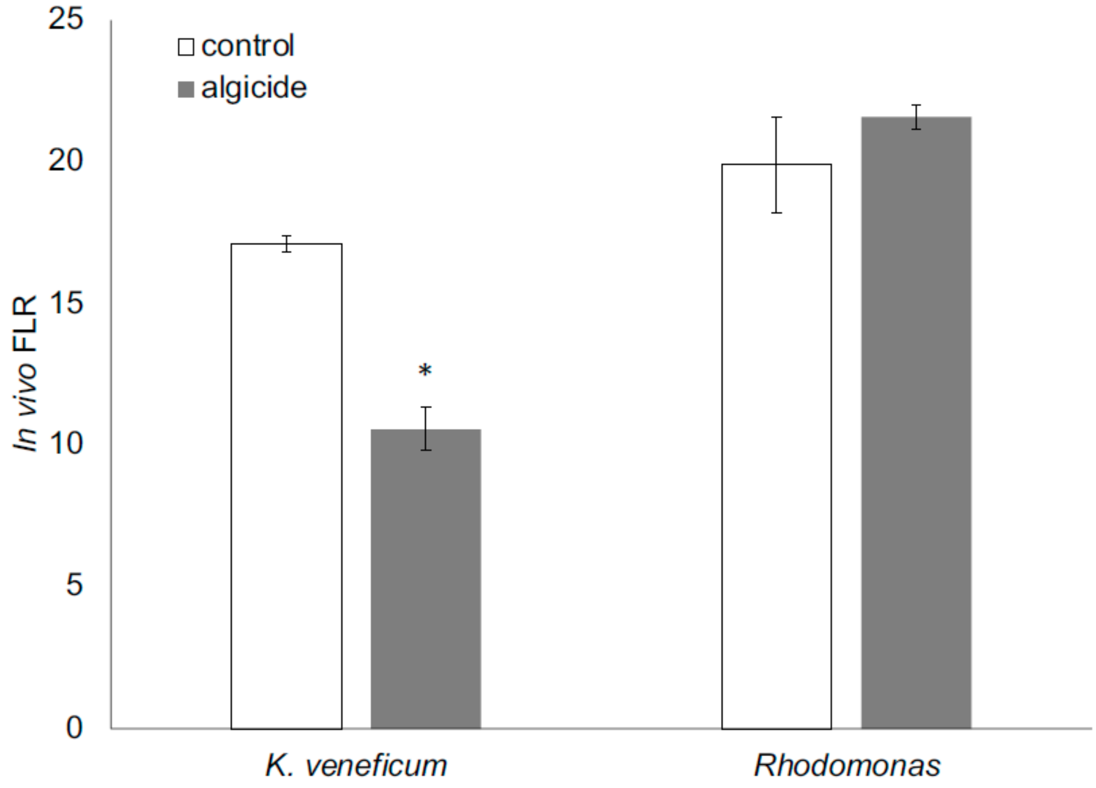

Supplementary Figure S1. In vivo fluorescence (FLR) of the harmful dinoflagellate *Karlodinium veneficum* and the control cryptophyte *Rhodomonas* treated with f/2 medium without the algicide (control) or the algicide for 19 h. Error bars represent standard deviations of 3 replicates. "*" indicates significant differences between the control and the treatment ($p < 0.05$).

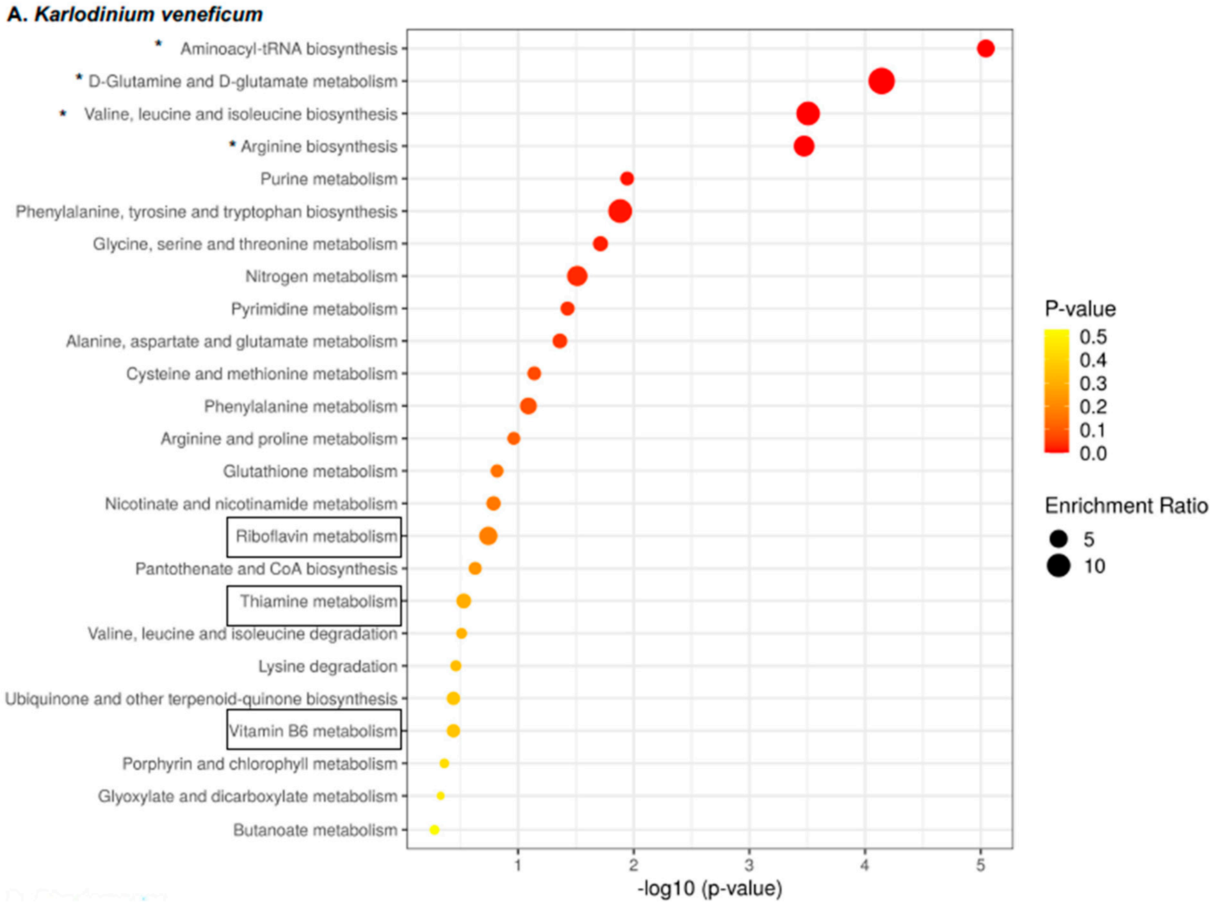

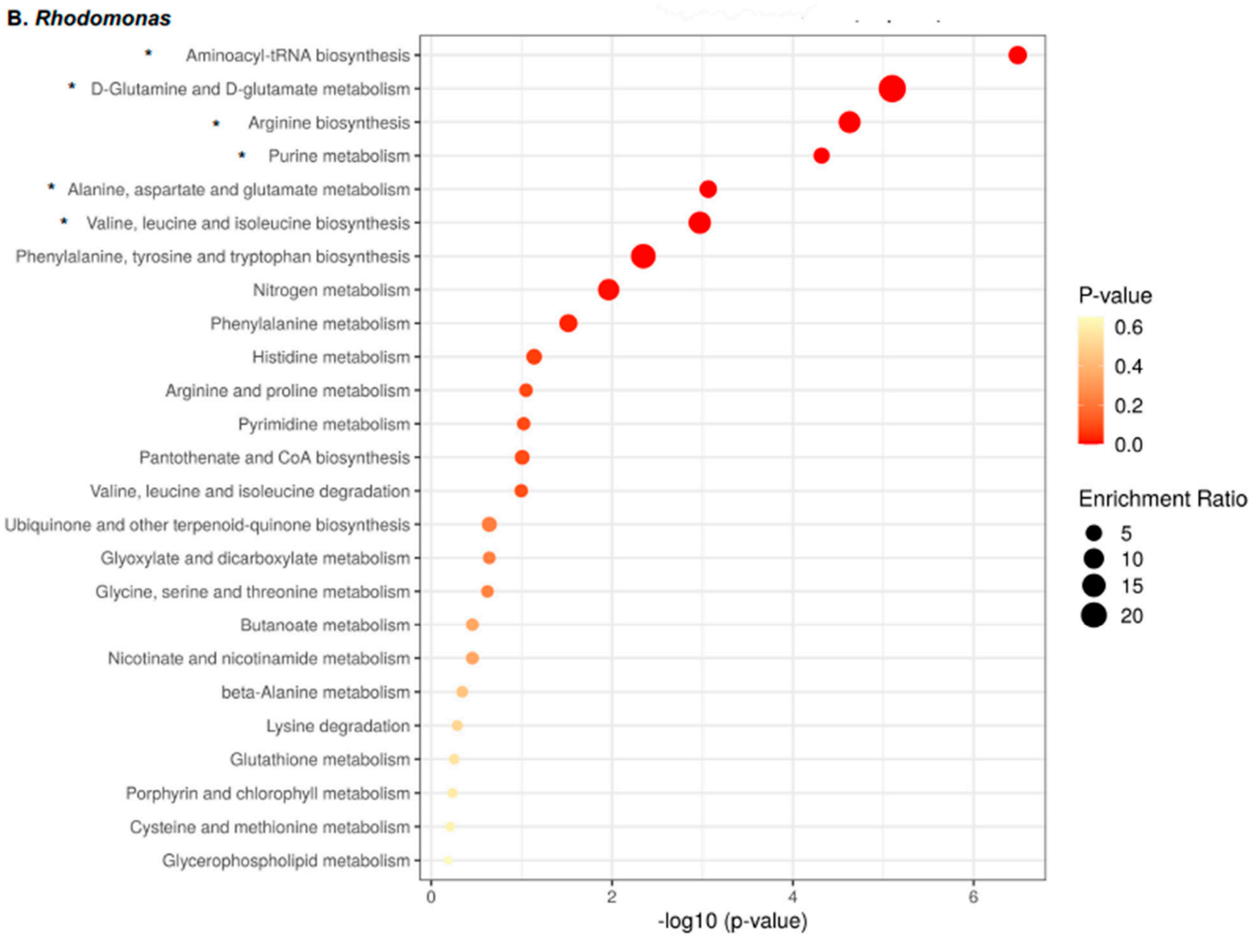

Supplementary Figure S2. Metabolite sets' enrichment analysis on metabolites increased (>1.5-fold comparing the treatment and the control) in cell pellets of *Karlodinium veneficum* (**A**) and *Rhodomonas* sp. (**B**).
